# Ethyl 6-(2-chloro­phen­yl)-4-methyl-1-(3-oxobut­yl)-2-thioxo-1,2,3,6-tetra­hydro­pyrimidine-5-carboxyl­ate

**DOI:** 10.1107/S160053680706730X

**Published:** 2008-01-04

**Authors:** Qing-Peng He, Jian-Yong Wang, Ruo-Kun Feng

**Affiliations:** a College of Chemistry and Chemical Engineering, Liaocheng University, Shandong 252059, People’s Republic of China

## Abstract

In the title mol­ecule, C_18_H_21_ClN_2_O_3_S, the pyrimidine ring exhibits a half-chair conformation. The ethyl group is disordered between two positions in a ratio 0.74:0.26. In the crystal structure, the mol­ecules are linked into chains along the *a* axis by N—H⋯O hydrogen bonds.

## Related literature

For the crystal structure of a related compound, see Jiang *et al.* (2007 ▶).
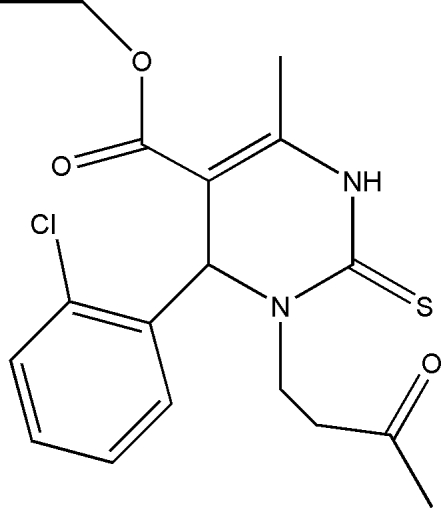


## Experimental

### 

#### Crystal data

C_18_H_21_ClN_2_O_3_S
*M* *_r_* = 380.88
Triclinic, 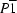

*a* = 7.5227 (12) Å
*b* = 9.7163 (15) Å
*c* = 14.122 (2) Å
α = 72.617 (6)°
β = 87.300 (6)°
γ = 71.296 (6)°
*V* = 931.7 (3) Å^3^
*Z* = 2
Mo *K*α radiation
μ = 0.34 mm^−1^
*T* = 273 (2) K
0.14 × 0.12 × 0.10 mm

#### Data collection

Bruker SMART CCD area-detector diffractometer
Absorption correction: multi-scan (*SADABS*; Sheldrick, 1996 ▶) *T*_min_ = 0.954, *T*_max_ = 0.967
10470 measured reflections
3233 independent reflections
2743 reflections with *I* > 2σ(*I*)
*R*_int_ = 0.021

#### Refinement

*R*[*F* ^2^ > 2σ(*F* ^2^)] = 0.055
*wR*(*F* ^2^) = 0.166
*S* = 1.00
3233 reflections
242 parameters
63 restraints
H-atom parameters constrained
Δρ_max_ = 0.92 e Å^−3^
Δρ_min_ = −0.71 e Å^−3^

### 

Data collection: *SMART* (Siemens, 1996 ▶); cell refinement: *SAINT* (Siemens, 1996 ▶); data reduction: *SAINT*; program(s) used to solve structure: *SHELXS97* (Sheldrick, 1997*a* ▶); program(s) used to refine structure: *SHELXL97* (Sheldrick, 1997*a* ▶); molecular graphics: *SHELXTL* (Sheldrick, 1997*b* ▶); software used to prepare material for publication: *SHELXTL*.

## Supplementary Material

Crystal structure: contains datablocks I, global. DOI: 10.1107/S160053680706730X/cv2372sup1.cif

Structure factors: contains datablocks I. DOI: 10.1107/S160053680706730X/cv2372Isup2.hkl

Additional supplementary materials:  crystallographic information; 3D view; checkCIF report

## Figures and Tables

**Table 1:** Hydrogen-bond geometry (Å, °)

*D*—H⋯*A*	*D*—H	H⋯*A*	*D*⋯*A*	*D*—H⋯*A*
N1—H1⋯O1^i^	0.86	2.16	2.984 (3)	160

Symmetry code: (i) 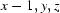.

**Fig. 1.**
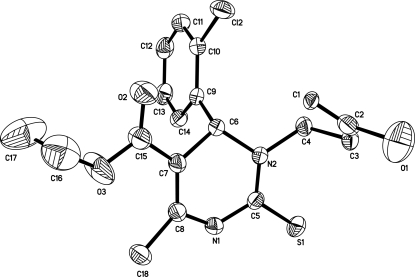
The molecular structure of (I) showing the atomic numbering and 30% probability displacement ellipsoids. Only major parts of disordered atoms is shown. H atoms omitted for clarity.

## supplementary crystallographic information

### Comment

Herewith we present the crystal strusture of the title compound, (I), which was synthesized through the reaction of 2-chlorobenzaldehyde and ethyl acetoacetate with urea derivative under solvent-free conditions.

In (I) (Fig. 1), bond lengths and angles are normal and comparable with those observed in reported compound (Jiang *et al.*, 2007). The pyrimidine ring exhibits a half-chair conformation with the maximal deviation of 0.168 Å for C6 from mean plane.

In the crystal, the molecules related by translation along axis a are linked into chains by N—H···O hydrogen bonds (Table 1).

### Experimental

2-Chlorobenzaldehyde (2 mmol), ethyl acetoacetate (2 mmol), urea derivatives (2.4 mmol) and H_3_BO_3_(0.4 mmol), in glacial acetic acid (10 ml) was heated at 373 K, while stirring for 1 h, then cooled to room temperature, and poured into ice water (50 ml), and recrystallized from EtOH, affording the title compound as a colourless crystalline solid. Elemental analysis: calculated for C_18_H_21_ClN_2_O_3_S: C 56.76, H 5.56, N 7.35%; found: C 56.68, H 5.45, N 7.44%.

### Refinement

All H atoms were placed in geometrically idealized positions (N—H 0.86, C—H 0.93–0.97 Å) and treated as riding on their parent atoms, with *U*_iso_(H) = 1.2 *U*_eq_(C, N). Atoms C16 and C17 were treated as disordered between two positions with refined occupancies of 0.740 (1) and 0.26 (1), respectively.

### Crystal data

**Table:**

C_18_H_21_ClN_2_O_3_S	*Z* = 2
*M**_r_* = 380.88	*F*_000_ = 400
Triclinic, *P*1	*D*_x_ = 1.358 Mg m^−^^3^
*a* = 7.5227 (12) Å	Mo *K*α radiation λ = 0.71073 Å
*b* = 9.7163 (15) Å	Cell parameters from 5002 reflections
*c* = 14.122 (2) Å	θ = 2.3–27.4º
α = 72.617 (6)º	µ = 0.34 mm^−^^1^
β = 87.300 (6)º	*T* = 273 (2) K
γ = 71.296 (6)º	Block, colourless
*V* = 931.7 (3) Å^3^	0.14 × 0.12 × 0.10 mm

### Data collection

**Table:**

Bruker SMART CCD area-detector diffractometer	3233 independent reflections
Radiation source: fine-focus sealed tube	2743 reflections with *I* > 2σ(*I*)
Monochromator: graphite	*R*_int_ = 0.021
*T* = 273(2) K	θ_max_ = 25.0º
φ and ω scans	θ_min_ = 1.5º
Absorption correction: multi-scan(SADABS; Sheldrick, 1996)	*h* = −8→8
*T*_min_ = 0.954, *T*_max_ = 0.967	*k* = −11→10
10470 measured reflections	*l* = −16→16

### Refinement

**Table:**

Refinement on *F*^2^	Secondary atom site location: difference Fourier map
Least-squares matrix: full	Hydrogen site location: inferred from neighbouring sites
*R*[*F*^2^ > 2σ(*F*^2^)] = 0.055	H-atom parameters constrained
*wR*(*F*^2^) = 0.166	*w* = 1/[σ^2^(*F*_o_^2^) + (0.0856*P*)^2^ + 1.0486*P*] where *P* = (*F*_o_^2^ + 2*F*_c_^2^)/3
*S* = 1.00	(Δ/σ)_max_ = 0.002
3233 reflections	Δρ_max_ = 0.92 e Å^−^^3^
242 parameters	Δρ_min_ = −0.71 e Å^−^^3^
63 restraints	Extinction correction: SHELXL97 (Sheldrick, 1997a), Fc^*^=kFc[1+0.001xFc^2^λ^3^/sin(2θ)]^-1/4^
Primary atom site location: structure-invariant direct methods	Extinction coefficient: 0.019 (5)

### Special details

**Table:**

Geometry. All e.s.d.'s (except the e.s.d. in the dihedral angle between two l.s. planes) are estimated using the full covariance matrix. The cell e.s.d.'s are taken into account individually in the estimation of e.s.d.'s in distances, angles and torsion angles; correlations between e.s.d.'s in cell parameters are only used when they are defined by crystal symmetry. An approximate (isotropic) treatment of cell e.s.d.'s is used for estimating e.s.d.'s involving l.s. planes.
Refinement. Refinement of *F*^2^ against ALL reflections. The weighted *R*-factor *wR* and goodness of fit *S* are based on *F*^2^, conventional *R*-factors *R* are based on *F*, with *F* set to zero for negative *F*^2^. The threshold expression of *F*^2^ > σ(*F*^2^) is used only for calculating *R*-factors(gt) *etc*. and is not relevant to the choice of reflections for refinement. *R*-factors based on *F*^2^ are statistically about twice as large as those based on *F*, and *R*- factors based on ALL data will be even larger.

### Fractional atomic coordinates and isotropic or equivalent isotropic displacement parameters (Å^2^)

**Table:**

	*x*	*y*	*z*	*U*_iso_*/*U*_eq_	Occ. (<1)
S1	0.25980 (13)	0.52300 (10)	0.37239 (6)	0.0576 (3)	
C1	0.7929 (7)	0.7860 (5)	0.2049 (3)	0.0787 (12)	
H1A	0.8645	0.7990	0.1467	0.094*	
H1B	0.8473	0.8105	0.2553	0.094*	
H1C	0.6657	0.8523	0.1885	0.094*	
O2	0.8603 (4)	0.1939 (5)	0.0855 (2)	0.0915 (11)	
O3	0.5995 (5)	0.2294 (6)	0.0050 (3)	0.1280 (14)	
N1	0.3010 (3)	0.4024 (3)	0.22578 (18)	0.0459 (6)	
H1	0.1819	0.4188	0.2294	0.055*	
N2	0.5778 (3)	0.3881 (3)	0.29602 (17)	0.0409 (6)	
O1	0.8827 (3)	0.5317 (3)	0.2018 (2)	0.0647 (7)	
C2	0.7951 (4)	0.6252 (4)	0.2424 (2)	0.0507 (7)	
C3	0.6906 (5)	0.5828 (4)	0.3335 (2)	0.0505 (7)	
H3A	0.5621	0.6511	0.3218	0.061*	
H3B	0.7468	0.5994	0.3878	0.061*	
C4	0.6868 (5)	0.4201 (3)	0.3659 (2)	0.0465 (7)	
H4A	0.8148	0.3511	0.3731	0.056*	
H4B	0.6334	0.3999	0.4305	0.056*	
C5	0.3905 (4)	0.4316 (3)	0.2963 (2)	0.0419 (6)	
C6	0.6877 (4)	0.2778 (3)	0.2446 (2)	0.0378 (6)	
H6	0.8002	0.3050	0.2211	0.045*	
C7	0.5756 (4)	0.2894 (3)	0.1548 (2)	0.0431 (7)	
C8	0.3869 (4)	0.3488 (3)	0.1493 (2)	0.0437 (7)	
C9	0.7520 (4)	0.1183 (3)	0.3179 (2)	0.0388 (6)	
C10	0.9389 (4)	0.0353 (3)	0.3472 (2)	0.0445 (7)	
C11	0.9927 (5)	−0.1094 (4)	0.4150 (3)	0.0569 (8)	
H11	1.1193	−0.1625	0.4324	0.068*	
C12	0.8590 (6)	−0.1737 (4)	0.4562 (3)	0.0592 (9)	
H12	0.8942	−0.2705	0.5020	0.071*	
C13	0.6714 (5)	−0.0942 (4)	0.4295 (3)	0.0574 (8)	
H13	0.5800	−0.1373	0.4575	0.069*	
C14	0.6196 (4)	0.0488 (4)	0.3613 (2)	0.0483 (7)	
H14	0.4928	0.1006	0.3438	0.058*	
C15	0.6932 (5)	0.2335 (5)	0.0802 (3)	0.0619 (9)	
C16	0.7016 (11)	0.2217 (13)	−0.0899 (6)	0.1280 (14)	0.740 (10)
H16A	0.6253	0.2918	−0.1489	0.154*	0.740 (10)
H16B	0.8211	0.2397	−0.0892	0.154*	0.740 (10)
C17	0.7244 (13)	0.0688 (10)	−0.0813 (8)	0.119 (3)	0.740 (10)
H17A	0.8124	0.0031	−0.0268	0.178*	0.740 (10)
H17B	0.7708	0.0476	−0.1417	0.178*	0.740 (10)
H17C	0.6055	0.0515	−0.0696	0.178*	0.740 (10)
C16'	0.719 (3)	0.101 (2)	−0.0367 (13)	0.124 (4)	0.260 (10)
H16C	0.8434	0.0499	−0.0034	0.148*	0.260 (10)
H16D	0.6574	0.0267	−0.0349	0.148*	0.260 (10)
C17'	0.725 (3)	0.195 (3)	−0.1337 (12)	0.119 (5)	0.260 (10)
H17D	0.6047	0.2722	−0.1532	0.179*	0.260 (10)
H17E	0.7526	0.1341	−0.1786	0.179*	0.260 (10)
H17F	0.8200	0.2412	−0.1354	0.179*	0.260 (10)
C18	0.2497 (5)	0.3678 (5)	0.0689 (3)	0.0608 (9)	
H18A	0.3170	0.3401	0.0144	0.073*	
H18B	0.1703	0.4721	0.0464	0.073*	
H18C	0.1740	0.3033	0.0946	0.073*	
Cl1	1.11696 (11)	0.11095 (11)	0.30061 (7)	0.0646 (3)	

### Atomic displacement parameters (Å^2^)

**Table:**

	*U*^11^	*U*^22^	*U*^33^	*U*^12^	*U*^13^	*U*^23^
S1	0.0587 (5)	0.0578 (5)	0.0593 (5)	−0.0108 (4)	0.0111 (4)	−0.0317 (4)
C1	0.097 (3)	0.060 (2)	0.081 (3)	−0.031 (2)	0.015 (2)	−0.019 (2)
O2	0.0477 (15)	0.158 (3)	0.0847 (19)	−0.0197 (17)	0.0138 (13)	−0.074 (2)
O3	0.0798 (19)	0.245 (4)	0.096 (2)	−0.039 (2)	0.0175 (16)	−0.120 (3)
N1	0.0346 (12)	0.0521 (15)	0.0538 (14)	−0.0099 (11)	0.0009 (10)	−0.0243 (12)
N2	0.0438 (13)	0.0361 (12)	0.0431 (12)	−0.0083 (10)	−0.0040 (10)	−0.0165 (10)
O1	0.0564 (14)	0.0666 (16)	0.0822 (17)	−0.0224 (12)	0.0144 (12)	−0.0371 (14)
C2	0.0461 (17)	0.0491 (18)	0.0602 (18)	−0.0144 (14)	−0.0057 (14)	−0.0209 (15)
C3	0.0572 (19)	0.0451 (17)	0.0556 (18)	−0.0152 (14)	−0.0027 (14)	−0.0247 (14)
C4	0.0529 (17)	0.0434 (16)	0.0436 (15)	−0.0117 (13)	−0.0077 (13)	−0.0163 (13)
C5	0.0437 (16)	0.0353 (14)	0.0448 (15)	−0.0093 (12)	0.0011 (12)	−0.0130 (12)
C6	0.0353 (14)	0.0370 (14)	0.0429 (14)	−0.0095 (11)	0.0003 (11)	−0.0164 (12)
C7	0.0427 (16)	0.0487 (17)	0.0416 (15)	−0.0152 (13)	0.0013 (12)	−0.0180 (13)
C8	0.0432 (16)	0.0476 (17)	0.0433 (15)	−0.0154 (13)	0.0017 (12)	−0.0169 (13)
C9	0.0405 (15)	0.0351 (14)	0.0444 (14)	−0.0097 (11)	0.0008 (11)	−0.0197 (12)
C10	0.0410 (15)	0.0432 (16)	0.0527 (17)	−0.0110 (12)	−0.0025 (12)	−0.0210 (13)
C11	0.0548 (19)	0.0435 (18)	0.065 (2)	−0.0028 (15)	−0.0165 (16)	−0.0176 (15)
C12	0.077 (2)	0.0376 (17)	0.0578 (19)	−0.0124 (16)	−0.0051 (17)	−0.0123 (14)
C13	0.069 (2)	0.0486 (19)	0.0612 (19)	−0.0262 (17)	0.0147 (16)	−0.0200 (16)
C14	0.0410 (16)	0.0449 (17)	0.0612 (18)	−0.0123 (13)	0.0065 (13)	−0.0216 (14)
C15	0.049 (2)	0.093 (3)	0.0526 (19)	−0.0214 (18)	0.0071 (15)	−0.0365 (19)
C16	0.0798 (19)	0.245 (4)	0.096 (2)	−0.039 (2)	0.0175 (16)	−0.120 (3)
C17	0.115 (5)	0.119 (6)	0.129 (7)	−0.028 (5)	0.036 (5)	−0.063 (5)
C16'	0.095 (6)	0.222 (8)	0.081 (6)	−0.025 (6)	0.020 (6)	−0.116 (6)
C17'	0.104 (9)	0.196 (10)	0.076 (8)	−0.034 (9)	0.028 (7)	−0.088 (8)
C18	0.0491 (19)	0.079 (2)	0.0567 (19)	−0.0157 (17)	−0.0071 (15)	−0.0273 (18)
Cl1	0.0387 (5)	0.0689 (6)	0.0853 (6)	−0.0185 (4)	−0.0042 (4)	−0.0190 (5)

### Geometric parameters (Å, °)

**Table:**

S1—C5	1.680 (3)	C8—C18	1.500 (4)
C1—C2	1.487 (5)	C9—C10	1.388 (4)
C1—H1A	0.9600	C9—C14	1.396 (4)
C1—H1B	0.9600	C10—C11	1.387 (5)
C1—H1C	0.9600	C10—Cl1	1.740 (3)
O2—C15	1.189 (4)	C11—C12	1.368 (5)
O3—C15	1.318 (5)	C11—H11	0.9300
O3—C16	1.523 (7)	C12—C13	1.381 (5)
O3—C16'	1.545 (10)	C12—H12	0.9300
N1—C5	1.371 (4)	C13—C14	1.378 (5)
N1—C8	1.382 (4)	C13—H13	0.9300
N1—H1	0.8600	C14—H14	0.9300
N2—C5	1.335 (4)	C16—C17	1.408 (11)
N2—C4	1.472 (4)	C16—H16A	0.9700
N2—C6	1.483 (3)	C16—H16B	0.9700
O1—C2	1.215 (4)	C17—H17A	0.9600
C2—C3	1.497 (5)	C17—H17B	0.9600
C3—C4	1.519 (4)	C17—H17C	0.9600
C3—H3A	0.9700	C16'—C17'	1.407 (13)
C3—H3B	0.9700	C16'—H16C	0.9700
C4—H4A	0.9700	C16'—H16D	0.9700
C4—H4B	0.9700	C17'—H17D	0.9600
C6—C7	1.512 (4)	C17'—H17E	0.9600
C6—C9	1.521 (4)	C17'—H17F	0.9600
C6—H6	0.9800	C18—H18A	0.9600
C7—C8	1.346 (4)	C18—H18B	0.9600
C7—C15	1.461 (4)	C18—H18C	0.9600
			
C2—C1—H1A	109.5	C10—C9—C14	116.3 (3)
C2—C1—H1B	109.5	C10—C9—C6	123.7 (3)
H1A—C1—H1B	109.5	C14—C9—C6	120.0 (2)
C2—C1—H1C	109.5	C11—C10—C9	122.3 (3)
H1A—C1—H1C	109.5	C11—C10—Cl1	117.0 (2)
H1B—C1—H1C	109.5	C9—C10—Cl1	120.8 (2)
C15—O3—C16	117.8 (4)	C12—C11—C10	119.8 (3)
C15—O3—C16'	109.7 (9)	C12—C11—H11	120.1
C16—O3—C16'	44.4 (9)	C10—C11—H11	120.1
C5—N1—C8	125.2 (2)	C11—C12—C13	119.6 (3)
C5—N1—H1	117.4	C11—C12—H12	120.2
C8—N1—H1	117.4	C13—C12—H12	120.2
C5—N2—C4	120.3 (2)	C14—C13—C12	120.1 (3)
C5—N2—C6	122.7 (2)	C14—C13—H13	120.0
C4—N2—C6	115.6 (2)	C12—C13—H13	120.0
O1—C2—C1	121.0 (3)	C13—C14—C9	121.9 (3)
O1—C2—C3	121.7 (3)	C13—C14—H14	119.0
C1—C2—C3	117.2 (3)	C9—C14—H14	119.0
C2—C3—C4	115.6 (3)	O2—C15—O3	121.8 (3)
C2—C3—H3A	108.4	O2—C15—C7	123.6 (3)
C4—C3—H3A	108.4	O3—C15—C7	114.6 (3)
C2—C3—H3B	108.4	C17—C16—O3	97.9 (7)
C4—C3—H3B	108.4	C17—C16—H16A	112.2
H3A—C3—H3B	107.4	O3—C16—H16A	112.2
N2—C4—C3	113.4 (2)	C17—C16—H16B	112.2
N2—C4—H4A	108.9	O3—C16—H16B	112.2
C3—C4—H4A	108.9	H16A—C16—H16B	109.8
N2—C4—H4B	108.9	C17'—C16'—O3	96.5 (10)
C3—C4—H4B	108.9	C17'—C16'—H16C	112.5
H4A—C4—H4B	107.7	O3—C16'—H16C	112.5
N2—C5—N1	116.0 (2)	C17'—C16'—H16D	112.5
N2—C5—S1	125.3 (2)	O3—C16'—H16D	112.5
N1—C5—S1	118.7 (2)	H16C—C16'—H16D	110.0
N2—C6—C7	110.4 (2)	C16'—C17'—H17D	109.5
N2—C6—C9	109.7 (2)	C16'—C17'—H17E	109.5
C7—C6—C9	113.2 (2)	H17D—C17'—H17E	109.5
N2—C6—H6	107.8	C16'—C17'—H17F	109.5
C7—C6—H6	107.8	H17D—C17'—H17F	109.5
C9—C6—H6	107.8	H17E—C17'—H17F	109.5
C8—C7—C15	126.8 (3)	C8—C18—H18A	109.5
C8—C7—C6	120.1 (3)	C8—C18—H18B	109.5
C15—C7—C6	113.1 (2)	H18A—C18—H18B	109.5
C7—C8—N1	118.3 (3)	C8—C18—H18C	109.5
C7—C8—C18	128.8 (3)	H18A—C18—H18C	109.5
N1—C8—C18	112.9 (3)	H18B—C18—H18C	109.5
			
O1—C2—C3—C4	−5.4 (4)	C7—C6—C9—C10	122.2 (3)
C1—C2—C3—C4	176.6 (3)	N2—C6—C9—C14	64.6 (3)
C5—N2—C4—C3	−79.4 (3)	C7—C6—C9—C14	−59.2 (3)
C6—N2—C4—C3	114.0 (3)	C14—C9—C10—C11	0.7 (4)
C2—C3—C4—N2	−67.4 (4)	C6—C9—C10—C11	179.3 (3)
C4—N2—C5—N1	177.4 (2)	C14—C9—C10—Cl1	−178.5 (2)
C6—N2—C5—N1	−17.0 (4)	C6—C9—C10—Cl1	0.1 (4)
C4—N2—C5—S1	−0.3 (4)	C9—C10—C11—C12	−0.8 (5)
C6—N2—C5—S1	165.4 (2)	Cl1—C10—C11—C12	178.4 (3)
C8—N1—C5—N2	−7.7 (4)	C10—C11—C12—C13	0.3 (5)
C8—N1—C5—S1	170.1 (2)	C11—C12—C13—C14	0.3 (5)
C5—N2—C6—C7	30.6 (4)	C12—C13—C14—C9	−0.4 (5)
C4—N2—C6—C7	−163.1 (2)	C10—C9—C14—C13	−0.1 (4)
C5—N2—C6—C9	−94.8 (3)	C6—C9—C14—C13	−178.7 (3)
C4—N2—C6—C9	71.4 (3)	C16—O3—C15—O2	−19.1 (9)
N2—C6—C7—C8	−22.2 (4)	C16'—O3—C15—O2	28.9 (11)
C9—C6—C7—C8	101.3 (3)	C16—O3—C15—C7	161.1 (6)
N2—C6—C7—C15	156.9 (3)	C16'—O3—C15—C7	−150.9 (10)
C9—C6—C7—C15	−79.7 (3)	C8—C7—C15—O2	173.4 (4)
C15—C7—C8—N1	−176.9 (3)	C6—C7—C15—O2	−5.5 (6)
C6—C7—C8—N1	2.0 (4)	C8—C7—C15—O3	−6.8 (6)
C15—C7—C8—C18	2.5 (6)	C6—C7—C15—O3	174.3 (4)
C6—C7—C8—C18	−178.6 (3)	C15—O3—C16—C17	107.2 (7)
C5—N1—C8—C7	15.2 (4)	C16'—O3—C16—C17	16.9 (14)
C5—N1—C8—C18	−164.4 (3)	C15—O3—C16'—C17'	−120.7 (15)
N2—C6—C9—C10	−114.0 (3)	C16—O3—C16'—C17'	−10.6 (11)

### Hydrogen-bond geometry (Å, °)

**Table:**

*D*—H···*A*	*D*—H	H···*A*	*D*···*A*	*D*—H···*A*
N1—H1···O1^i^	0.86	2.16	2.984 (3)	160

Symmetry codes: (i) *x*−1, *y*, *z*.

## References

[bb1] Jiang, H., Yu, C.-X., Tu, S.-J., Wang, X.-S. & Yao, C.-S. (2007). *Acta Cryst.* E**63**, o298–o299.

[bb2] Sheldrick, G. M. (1996). *SADABS* University of Göttingen, Germany.

[bb3] Sheldrick, G. M. (1997*a*). *SHELXS97* and *SHELXL97* University of Göttingen, Germany.

[bb4] Sheldrick, G. M. (1997*b*). *SHELXTL.* Version 5.1. Bruker AXS Inc., Madison, Wisconsin, USA.

[bb5] Siemens (1996). *SMART* and *SAINT* Siemens Analytical X-ray Instruments Inc., Madison, Wisconsin, USA.

